# Influence of Skin–Core Stiffness Mismatch on the Static and Dynamic Mechanical Performance of CFRP- and QFRP-Skinned Aramid Honeycomb Sandwich Structures

**DOI:** 10.3390/polym18172090

**Published:** 2026-08-28

**Authors:** Madalina Andreea Mustareata, Raluca Maier, Teodor Adrian Badea, Alexandru Ciubotariu, Andrei Timonia, Vlad Buga, Laurentiu Petre, Ciprian Ionuț Morăraș, Viorel Goanță

**Affiliations:** 1 Composite Materials Laboratory for Aeronautical Field, Romanian Research and Development Institute for Gas Turbines—COMOTI, 220D Iuliu Maniu Av., 061126 Bucharest, Romania; madalina.mustareata@comoti.ro (M.A.M.); teodor.badea@comoti.ro (T.A.B.); alexandru.ciubotariu@comoti.ro (A.C.); andrei.timonia@comoti.ro (A.T.); vlad.buga@comoti.ro (V.B.); laurentiu.petre@comoti.ro (L.P.); 2 Mechanical Engineering, Mechatronics and Robotics Department, Mechanical Engineering Faculty, “Gheorghe Asachi” Technical University of Iasi, 700050 Iasi, Romania; ciprian-ionut.moraras@academic.tuiasi.ro (C.I.M.); viorel.goanta@academic.tuiasi.ro (V.G.)

**Keywords:** sandwich structure, composite, Nomex core, Flex-Core, flatwise compression, flexural tests, DMA (Dynamic Thermo-Mechanical Analysis)

## Abstract

Valued for their lightweight, high strength-to-weight ratio, and energy absorption, sandwich composites were adopted across multiple industries. This paper evaluates the static and dynamic mechanical performances of sandwich configurations exploiting carbon fiber-reinforced polymer (CFRP) and quartz fiber-reinforced polymer (QFRP) composite skins bonded to hexagonal Nomex^®^ or compliant Flex-Core^®^ cores. Skin thickness varied negligibly; therefore, this study focused on skin type and particularly core architecture influence on mechanical behavior. Three-point bending and flatwise compression tests evaluated flexural stiffness, core shear strength, and core compressive strength. DMA analysis was used to characterize their temperature-dependent viscoelastic response through the storage modulus, loss modulus, and damping factor. QFRP/Nomex emerged as the optimal configuration achieving the highest flexural stress, outperforming CFRP/Nomex by 14%, while restricting strain to 2.4% (compared to 7% for CFRP). DMA (Dynamic Mechanical Analyzer) analysis showed that QFRP/Nomex exhibits the highest storage and loss moduli. Observing the energy at break, CFRP/Nomex^®^ sandwiches stand out in their ability to absorb 60% more energy than QFRP/Nomex^®^ before total failure occurs, showing an overall superior energy absorption of CFRP skins. Conversely, flatwise compression tests revealed that QFRP/Flex-Core^®^ excelled in yield and compressive strengths, outperforming CFRP/Nomex by 10% and 8%, respectively, due to superior elastic matching. DMA damping profiles confirmed that the geometric compliance of curved Flex-Core^®^ cell walls in conjunction with QFRP skins accelerates structural yielding under shear and viscoelastic energy dissipation prior to chemical softening. This work highlights that sandwich structure design critically depends on managing core architecture and skin-to-core stiffness mismatch.

## 1. Introduction

Comprising a central core bonded between two thin, stiff face sheets, sandwich structures represent a leading weight-optimized structural solution, with specific bending stiffness and strength-to-weight performance substantially exceeding the capabilities of equivalent-mass monolithic laminates [1]. Among available core materials, including polymeric foams, metallic foams, balsa wood, and corrugated sheets, Nomex honeycomb has become the preferred solution for aerospace structures, owing to its low density, high specific stiffness, good thermal stability, and flame resistance [2,3]. Consequently, Nomex sandwich panels are extensively used in aircraft floors, doors, wing flaps, fairings, nacelles, spoilers, engine cowls, and interior panels [4,5]. The mechanical behavior of Nomex honeycomb sandwich structures has been extensively investigated under three-point bending tests [6,7], flatwise compression [7,8], fatigue loading [9,10], and impact loading [11]. Its thermo-mechanical response and degradation under coupled loading conditions have also been investigated [12], demonstrating that temperature-dependent effects significantly modify effective stiffness and shear response. These studies demonstrate that the overall structural response depends on the combined behavior of the skins and honeycomb core, with the skins carrying most of the bending and in-plane loads, while the core provides transverse shear stiffness and stabilizes the structure against buckling [7]. Furthermore, recent studies have demonstrated that honeycomb sandwich panels can absorb more energy than equivalent-mass solid or stiffened structures and that the geometry and dimensions of the honeycomb cells have significant impacts on the structural efficiency [13].

Beyond conventional hexagonal honeycombs, alternative architectures such as over-expanded and flexible (Flex-Core) honeycombs have also been developed to improve bending compliance while maintaining adequate structural performance and enabling multifunctional applications [14]. While conventional hexagonal honeycombs have been extensively investigated, the mechanical behavior of Flex-Core honeycomb sandwich structures remains comparatively unexplored, particularly when combined with composite face sheets.

The structural performance of Nomex honeycomb panels relies mainly on skin-to-core interaction and bonding, rather than on the core alone. Therefore, since the skins bear most of the in-plane and bending loads, their selection strongly influences overall stiffness, strength, fatigue life, damage tolerance, and functional performance. Carbon fiber-reinforced polymer (CFRP) skins remain a primary choice in Nomex honeycomb sandwich structures due to their exceptional specific stiffness, strength, and fatigue resistance [1,15], whereas glass fiber-reinforced polymer (GFRP) offers improved ductility and lower cost, particularly in hybrid laminates where glass or Kevlar fibers enhance energy absorption, damage tolerance, and deformation capability [1]. Nevertheless, these studies primarily focus on conventional structural skins, leaving less attention to specialized fiber systems that may provide additional functional advantages.

Quartz fiber-reinforced polymer (QFRP) represents a specialized alternative for electromagnetic–transparent structures such as aircraft radomes, combining good mechanical performance with low dielectric constant and dielectric loss [16,17]. Hybrid skin structures (carbon, glass, and Kevlar fibers in different stacking configurations) and Nomex honeycomb sandwich panels’ mechanical performances [1] were reported, highlighting that skin thickness and fiber composition significantly affect the flexural strength, modulus, and toughness. Glass or Kevlar fiber content exhibited better ductility and energy absorption, while carbon-dominated configurations demonstrated higher strength at the expense of lower deformation capacity. Quartz fibers, composed of amorphous high-purity silica, exhibit a low and thermally stable dielectric constant and very low dielectric loss, properties that make them uniquely suited to electromagnetic transparency applications in which CFRP would be prohibitive due to its electrical conductivity and GFRP sub-optimal due to its higher permittivity. For example, radomes, the aerodynamic enclosures protecting the radar, impose simultaneous structural and electromagnetic performance requirements that have made honeycomb sandwich panels with quartz fiber composite skins the construction standard for airborne radome structures [16,17]. However, compared with the extensive literature available for CFRP and GFRP skins, the mechanical and thermo-mechanical behavior of QFRP/Nomex sandwich configurations remains comparatively less investigated, particularly when the influence of skin material is considered together with the core architecture and adhesive interface.

Sandwich structure cores (e.g., Nomex honeycomb) are typically manufactured by secondary bonding, co-bonding, or co-curing, the latter being the preferred aerospace process because it simultaneously consolidates and bonds the entire assembly in a single autoclave cycle. Nevertheless, co-curing remains sensitive to adhesive flow, prepreg consolidation, and cure kinetics, which may generate voids, insufficient adhesive filets, and poor wetting at the skin-core interface, ultimately affecting load transfer and structural reliability [18,19,20].

Although significant studies have been conducted on Nomex honeycomb sandwich structures, key aspects remain insufficiently addressed. While conventional hexagonal cores with CFRP skins are extensively studied, different architectures and less common skin materials have been insufficiently explored. For example, Flex-Core configurations are scarcely studied experimentally, especially with composite face sheets, and QFRP/Nomex sandwich structures are less investigated than CFRP ones. Furthermore, there is a lack of systematic comparative research on various core architectures coupled with CFRP and QFRP skins under uniform conditions, despite their influence on load transfer, deformation behavior, and overall structural response.

To address this gap, this study experimentally investigates Flex-Core and conventional Nomex sandwich structures with CFRP and QFRP skins, evaluating their flexural, compressive, and thermo-mechanical response, including viscoelastic behavior under cyclic loading.

Consequently, this work systematically investigates how skin materials and core architectures affect the flexural and compressive strength of honeycomb sandwich composites. DMA analysis provides insights into the combined response of the skins, core, and adhesive interfaces, specifically evaluating overall bending stiffness, energy absorption capability, viscoelastic response, skin-to-core bond integrity, and composite shear behavior under standard, low-frequency (1 Hz) cyclic loading across a specific temperature range. Skin thickness varied negligibly since only two plies of QFRP or CFRP prepreg, both roughly 0.28 mm thick, were applied using similar curing and vacuum bagging techniques. The results were comparatively evaluated under the same testing conditions. Overall, the study offers an application-oriented experimental contribution, highlighting the significant influence of skin and core selection and skin-to-core stiffness mismatch on the structural integrity and mechanical reliability of aramid-core composite skin sandwich structures for advanced engineering applications.

## 2. Materials and Methods

### 2.1. Materials

The choice of the core materials was driven by the specific request in various aeronautic applications to develop ultra-lightweight and extremely rigid sandwich structures adapted for complex shapes. Nomex is a lightweight, heat-resistant material with low thermal conductivity. Despite being lightweight, Nomex maintains good mechanical strength. As mentioned above, owing to its exceptional strength and durability, it has made significant inroads into the aerospace industry, where it is utilized in applications such as aircraft components, structures, and insulation.

Nevertheless, although Nomex honeycomb is highly rigid and orthotropic, when forced onto tight radii, it suffers from anticlastic curvature (saddle-shaping) and cell-wall buckling. By contrast, HexWeb^®^ Nonmetallic Flex-Core^®^ honeycomb (Hexcel Corporation, Casa Grande, AZ, USA) developed based on Flex-Core technologies solves this by altering the cell geometry to allow multi-axis bending without cell distortion. Thus, the core materials of the developed sandwich structures within the present work were HexWeb^®^ HRH-10-3.2-64 Nomex^®^ aramid-paper/phenolic honeycomb (Hexcel Composites Limited, Duxford, UK) and HexWeb^®^ Nonmetallic Flex-Core^®^, both made out of DuPont™ Nomex^®^ aramid paper (DuPont, Richmond, VA, USA) dipped in phenolic resin aramid HRH10 aeronautic grade, with a 6 ± 0.1 mm thickness and both purchased from Hexcel Corporation (Stamford, CT, USA). HexWeb^®^ Nonmetallic Flex-Core^®^ HRH10-F50 (Hexcel Corporation, Casa Grande, AZ, USA),with anumber of cells per 305 mm measured along the W-direction, had a density of 56.1 kg/m^3^, while Nomex^®^ HRH-10-3.2-64 had a hexagonal cell size of 3.2 mm and a density of 64 kg/m^3^. While Nomex honeycomb cores utilize rigid, flat-walled hexagonal shapes, Flex-Core^®^ relies on highly unique curved, corrugated cell walls that distinctively resemble a series of bells.

**Figure 1:**
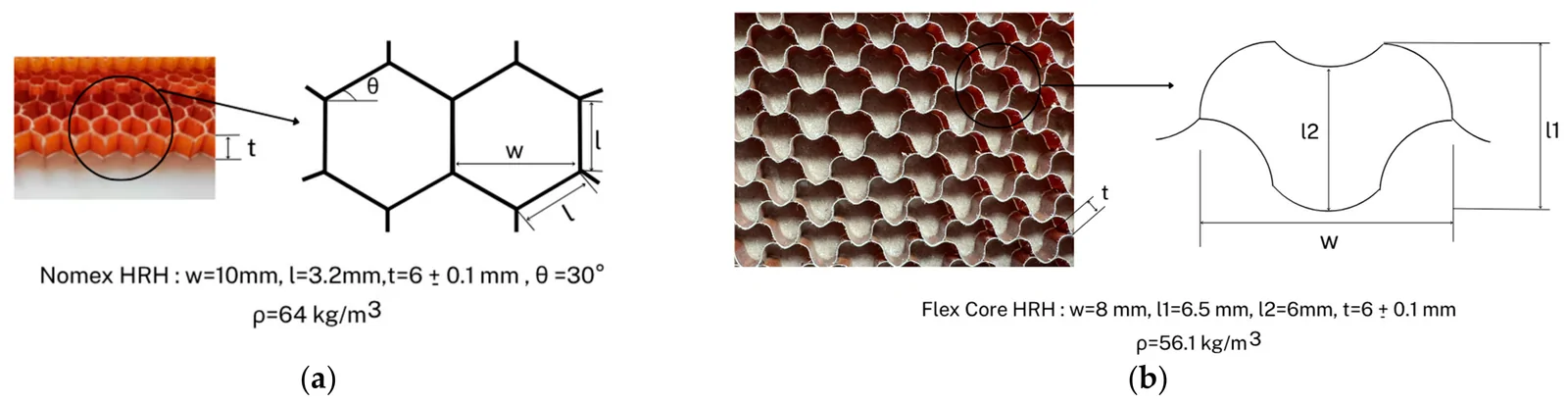
Geometry and parameters of core materials under study: (**a**) Nomex^®^ honeycomb; (**b**) Flex-Core^®^.

Further in the study, the core architecture was one of the key parameters to investigate during the sandwich structures assessment from a mechanical perspective. Figure 1 shows the geometry of the core materials under study: Figure 1a shows the Nomex^®^ honeycomb; Figure 1b shows Flex-Core^®^. These figures depict the geometric and dimensional features of the two investigated core materials.

For this proposed work, two different types of composite fabric were selected for the fabrication of laminate skins. These include pre-impregnated carbon and quartz woven fiber: HexPly^®^ M49/42%/245 g/T2 × 2/CHS-3k, purchased from Hexcel, and EX-1515 8HS 4581 AQ III, 330 g/m^2^, purchased from Toray. The carbon-reinforced prepreg is a high-strength carbon (CHS-3K with a nominal areal weight of 200 g/m^2^ and a twill 2 × 2 woven) epoxy (M49 42%wt., nominal density 1.18 g/cm^3^) system. The quartz-reinforced prepreg is a quartz (Astroquartz^®^ III 4581 (AQIII) with a nominal areal weight of 300 g/m^2^ and an 8HSatin woven) cyanate ester (Toray EX-1515 40%wt.) system.

The skin materials were chosen with the aim of addressing sandwich configurations suitable for aerospace use, not just investigating their strength-to-weight performances (CFRP) but also their applications requiring exceptional wave transparency (low dielectric constant and loss tangent), high-temperature stability, and low thermal expansion (QFRP). The interfaces were created by an interlayer adhesive film integration between the skin and the core: Toray MicroPly™ TC310 toughened epoxy for CFRP configuration and MicroPly™ EX-1516 Film-cyanate ester for QFRP configuration. Both adhesive films were chosen due to their chemical compatibility with the main skin polymer systems as well as for their excellent mechanical properties, good resistance to moisture, and toughness. The nominal thickness of the adhesive films used was 60 µm (MicroPly™ EX-1516 Film) and 150 µm (MicroPly™ TC310). Table 1 below outlines the characteristics of the two cores selected for this study.

Therefore, the developed sandwich structures consist of two thin laminates, stiff outer skins (such as carbon fiber or quartz) bonded to an overall 6 mm thick lightweight core structure: cellular hexagonal (Nomex) or a bell-shaped geometry (Flex-Core). These composite sandwich structures leverage strength and low weight, resulting in products with increased durability, mechanical resistance, and reduced weight.

It should be noted that the CFRP and QFRP configurations represent different material systems, since the CFRP skins employed an epoxy matrix and TC310 adhesive, whereas the QFRP skins employed a cyanate ester matrix and EX-1516 adhesive. Therefore, the present comparison does not isolate the effect of fiber type alone, and differences in matrix, adhesive, and fiber architecture may also contribute to the observed mechanical and thermo-mechanical response.

#### Process-Sample Manufacturing

**Figure 2:**
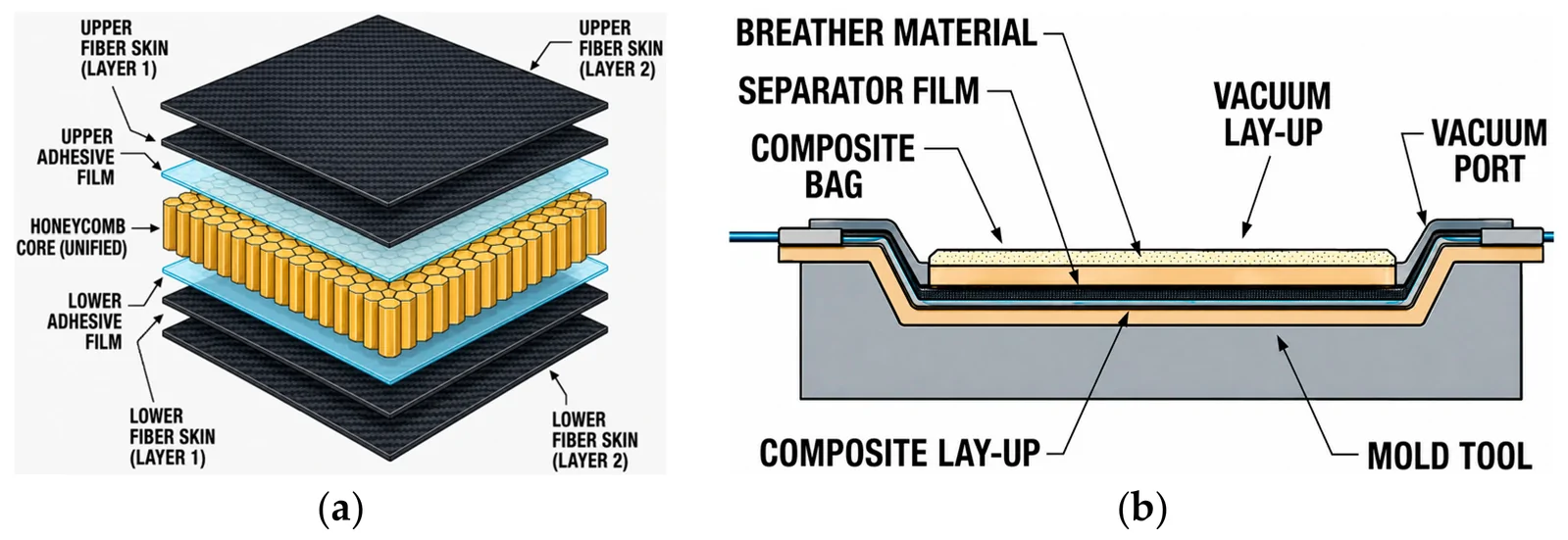
Sandwich manufacturing: (**a**) exploded view of the sandwich layers; (**b**) vacuum bagging process assembly.

**Figure 3:**
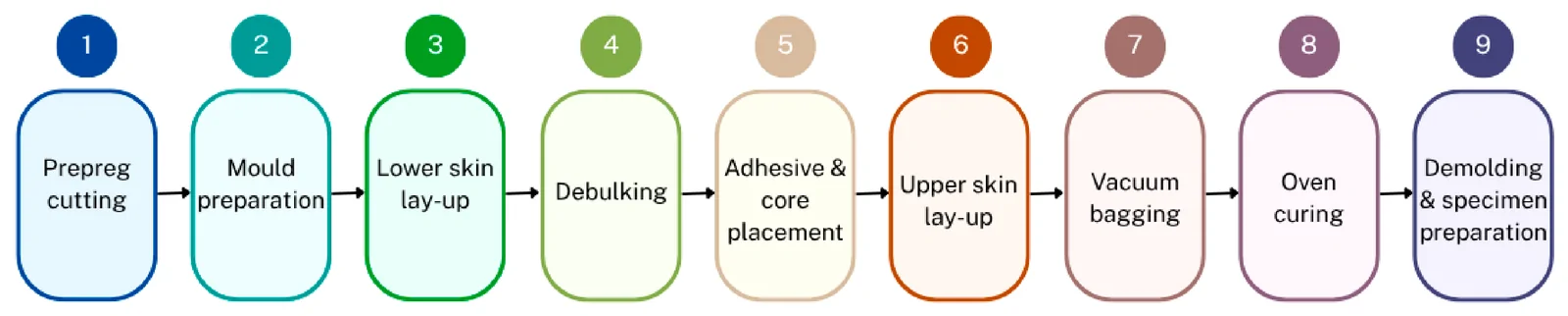
Workflow of the samples manufacturing process: oven vacuum-assisted bagging.

The samples were manufactured at room temperature by hand lay-up of prepreg coupons cut using a Eastman Eagle S125 static-table CNC cutting system (60 in × 8 ft; Eastman Machine Company, Buffalo, NY, USA) to the dimensions required for each testing geometry. A one-step manufacturing process was adopted for all the investigated configurations, in which the skins, adhesive films, and honeycomb core were assembled and cured simultaneously; thus, the skins were not pre-cured separately, aiming to streamline the process and cut costs. The cut plies were then placed on a metallic plate mold previously coated with a release agent and stacked in a 0° orientation sequence (lower skin). The laminate was debulked at −0.9 bar vacuum. The adhesive film and the core were placed on the lower skin, followed by the adhesive film and the corresponding prepreg plies for the upper skin, using the same stacking procedure. After creating the sandwich structural assembly, the vacuum bagging process was performed. For that, a sheet of release film and a breather layer were added on top of the assembly and were then enclosed in a vacuum bag to create a vacuum-sealed environment. This step is crucial for preventing the formation of any voids and for minimizing the exposure of the epoxy to air during the curing process. Two vacuum lines were used: one for creating the vacuum and the other for measuring during the curing cycle. The mold/sample vacuum bag assembly was placed inside an oven for polymerization and curing under the requisite temperature while maintaining the vacuum contact to 900 mbar. Figure 2 shows the sandwich architecture and the vacuum bagging assembly, and Figure 3 shows the workflow of the main manufacturing stages.

**Figure 4:**
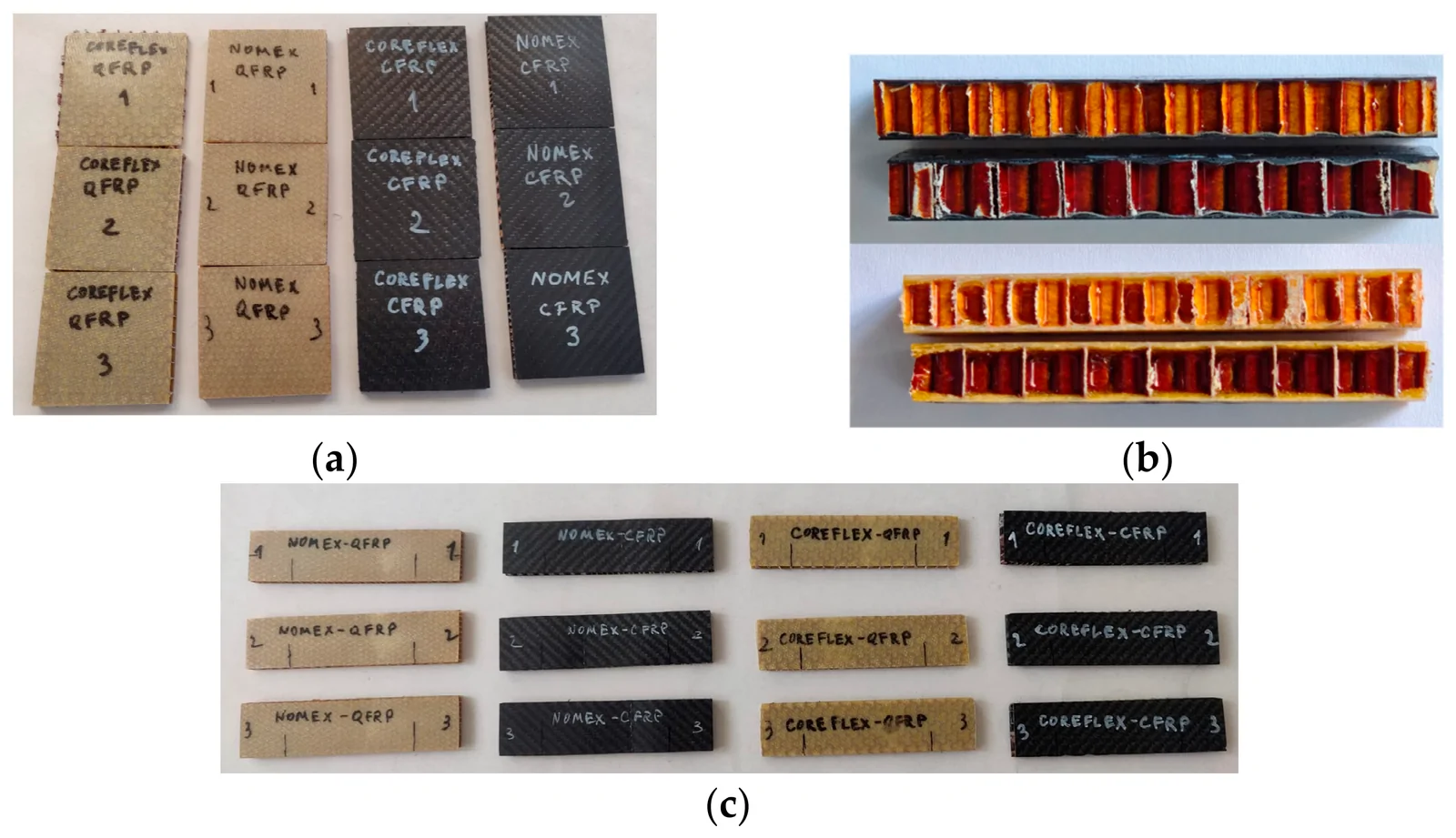
Sandwich specimens subjected to experimental investigation: (**a**) samples for flatwise compression test; (**b**) sample section view; and (**c**) samples for 3-point bending test.

Since both epoxy and cyanate ester polymeric systems share the same activation temperature, the curing cycle was nearly similar for both skin materials, with the exception of dwell exposure times, which are directly related to the adhesive film bonding system. The one-stage curing cycle applied started with a heat-up (3 °C/min heating rate), raising the temperature to 120 °C, dwell/hold at 120 °C for 120 min (CFRP skin sandwich) and 300 min (QFRP skin sandwich), followed by cooling down to 60 °C (3 °C/min), then removed from the metallic plate mold. The post-processing of the manufactured sandwich structures was not laborious, since a fine grinding wheel disk cutter with the minimum thickness was used for slightly adjusting sandwich composite structures, without damaging the fibers or the core. Four sandwich configurations were therefore manufactured, combining two face-sheet materials (CFRP and QFRP) with two honeycomb core architectures (conventional hexagonal Nomex and Flex-Core honeycomb). The resulting specimens used for the experimental investigation are illustrated in Figure 4.

### 2.2. Experimental Setup

#### 2.2.1. Compression Tests

The mechanical response of the sandwich composite structures under compression was evaluated using an Instron 8801 universal testing machine with a maximum load capacity of 100 kN. Compression tests were performed in accordance with the ASTM C365 standard [21]. The tests were performed at room temperature (laboratory atmosphere 23 ± 3 °C, 50 ± 5% relative humidity) under displacement control at a crosshead speed of 0.5 mm/min (except QFRP-1-Flex-Core^®^ tested under 2 mm/min), until specimen failure. Compression test samples were square, and dimensions were 60 × 60 mm (cross-sectional area 3600 mm^2^), the depth of the specimen being equal to the thickness of the sandwich construction (dimensions are given in Table 2). For each type of investigated sandwich configuration, 3 specimens were prepared and tested.

#### 2.2.2. Three-Point Bending Tests

**Figure 5:**
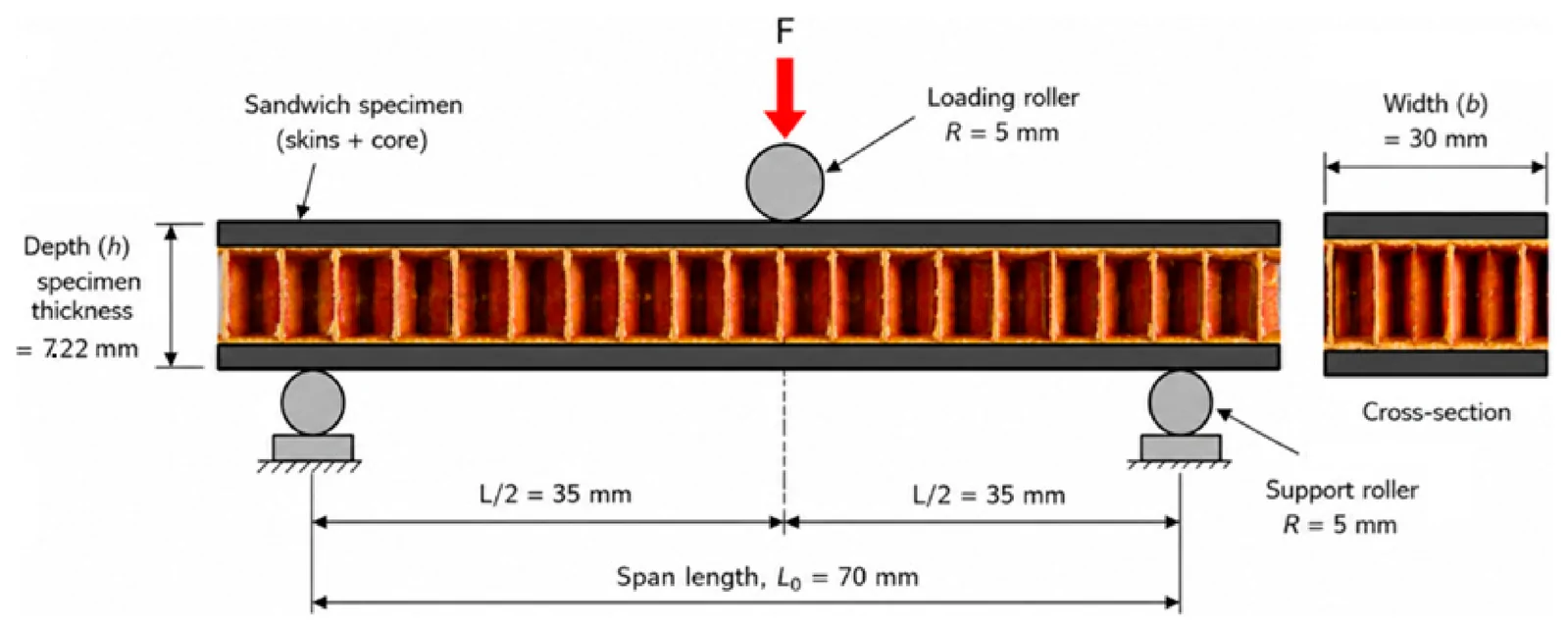
Schematic representation of the three-point bending test configuration according to ASTM C393/C393M.

The core shear properties of sandwich constructions evaluated by three-point static bending tests were performed in accordance with the ASTM C393/C393M standard [23]. This test minimizes the influence of bending stress in the skin under bending load, and the specimen undergoes shear failure or compression failure of the core material to test the shear failure resistance of the sandwich panel. Three-point bending tests were conducted on an Instron 34SC-5 universal testing machine, manufactured by Instron ITW—Illinois Tool Works from Norwood, MA, USA, equipped with a 5 kN load cell. The test specimen geometry was rectangular in cross section, with a width of 30 mm and a length of 120 mm, the depth of the specimen being equal to the thickness of the sandwich construction. The tests were performed at room temperature (laboratory atmosphere 23 ± 3 °C, 50 ± 5% relative humidity). For testing, the sample was placed on the three-point bend test fixture, and the span length was adjusted to 70 mm, as shown in Figure 5. The radius of loading rollers was 5 mm. The samples were tested at a crosshead displacement of 6 mm/min. until specimen failure or until a deflection equal to the specimen thickness is reached.

The purpose of these tests was to determine the mechanical performance (bending resistance, bending modulus of elasticity, and other aspects linked to the effort–deformation relationship) of the sandwich structures fabricated. Table 2 summarizes the materials, geometry, and dimension features of the sandwich structure specimens tested in static regimes (under compression and three-point bending).

#### 2.2.3. Dynamic Mechanical Analyzer (DMA)

Dynamic Mechanical Analyzer (DMA) was performed using a TA Instruments DMA 850 analyzer (New Castle, DE, USA) operating in three-point bending mode at an oscillation frequency of 1 Hz in accordance with ASTM D7028-7:2015 [22]. The temperature sweep was conducted from room temperature up to 160 °C at a heating rate of 5 ± 1 °C/min. The instrument provides a modulus accuracy of ±1%, with isothermal stability better than ±0.1 °C above 50 °C. The tan δ resolution and sensitivity are 1 × 10^−5^ and 1 × 10^−4^, respectively. Rectangular specimens with dimensions of 59 ± 1 mm × 12 ± 1 mm × 6.5 ± 0.5 mm were tested. Three samples were analyzed for each configuration.

## 3. Results

### 3.1. Mechanical Properties

The mechanical performance of the four sandwich configurations was evaluated through flatwise compression, three-point bending, and dynamic mechanical analysis (DMA). The compression and bending tests were used to assess the load-bearing behavior and deformation response of the structures, while DMA was used to characterize their temperature-dependent viscoelastic behavior. The results are presented and compared in the following sections, with particular emphasis on the effects of skin material and core architecture.

### 3.2. Compression Results

**Figure 6:**
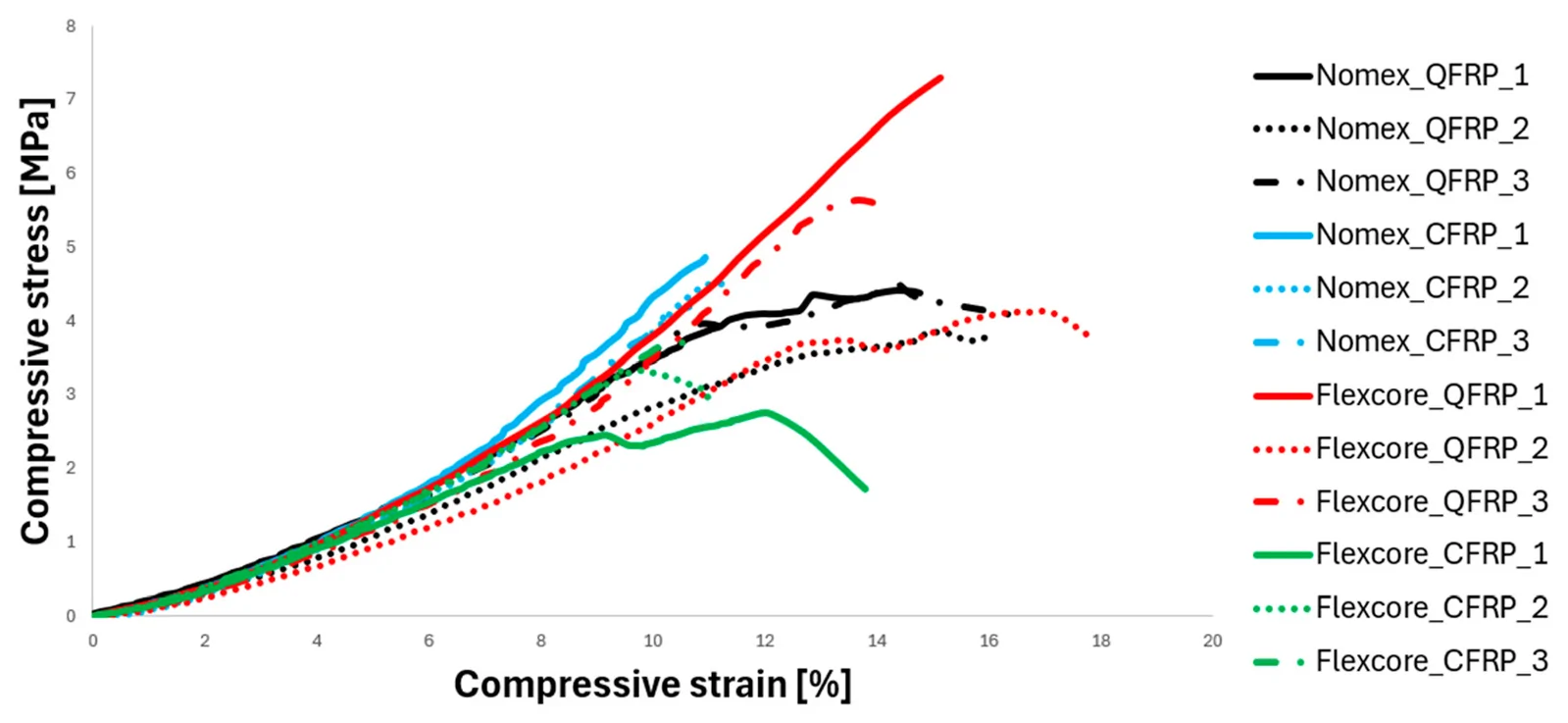
Compression stress–strain curves for all developed sandwich configurations.

In Out-of-Plane (Flatwise) compression, a compressive load is applied perpendicular to the panel surfaces (crushing the sandwich), the strength being dictated by the material, density, and cell configuration of the core. The stiffness of CFRP or QFRP skins distributes the load, while the interface plays an important role too. The compression stress–strain curves summarizing all tested sandwich configurations are given in Figure 6. The highest maximum compression stress (7.3 MPa) and strain (15%) were recorded for the QFRP-Flex-Core^®^-1 specimen tested at a higher speed rate (2 mm/min), excluded from further analysis.

Different mechanical responses can be observed among the sandwiches tested at 0.5 mm/min, reflecting the influence of the skin material, core geometry, and buckling mechanism. In all four cases, the nearly identical initial slopes indicate that all sandwich configurations exhibit comparable elastic behavior under compression. The divergence of the curves beyond approximately 6–7% strain suggests that the influence of the skin material and core architecture becomes significant only after damage initiation within the sandwich structure. The highest mean compression stress was obtained for the QFRP-Flex-Core^®^ configuration (4.9 MPa, with a maximum value of 5.6 MPa), followed by the CFRP-Nomex^®^ configuration (4.5 MPa, with a maximum value of 4.9 MPa).

**Figure 7:**
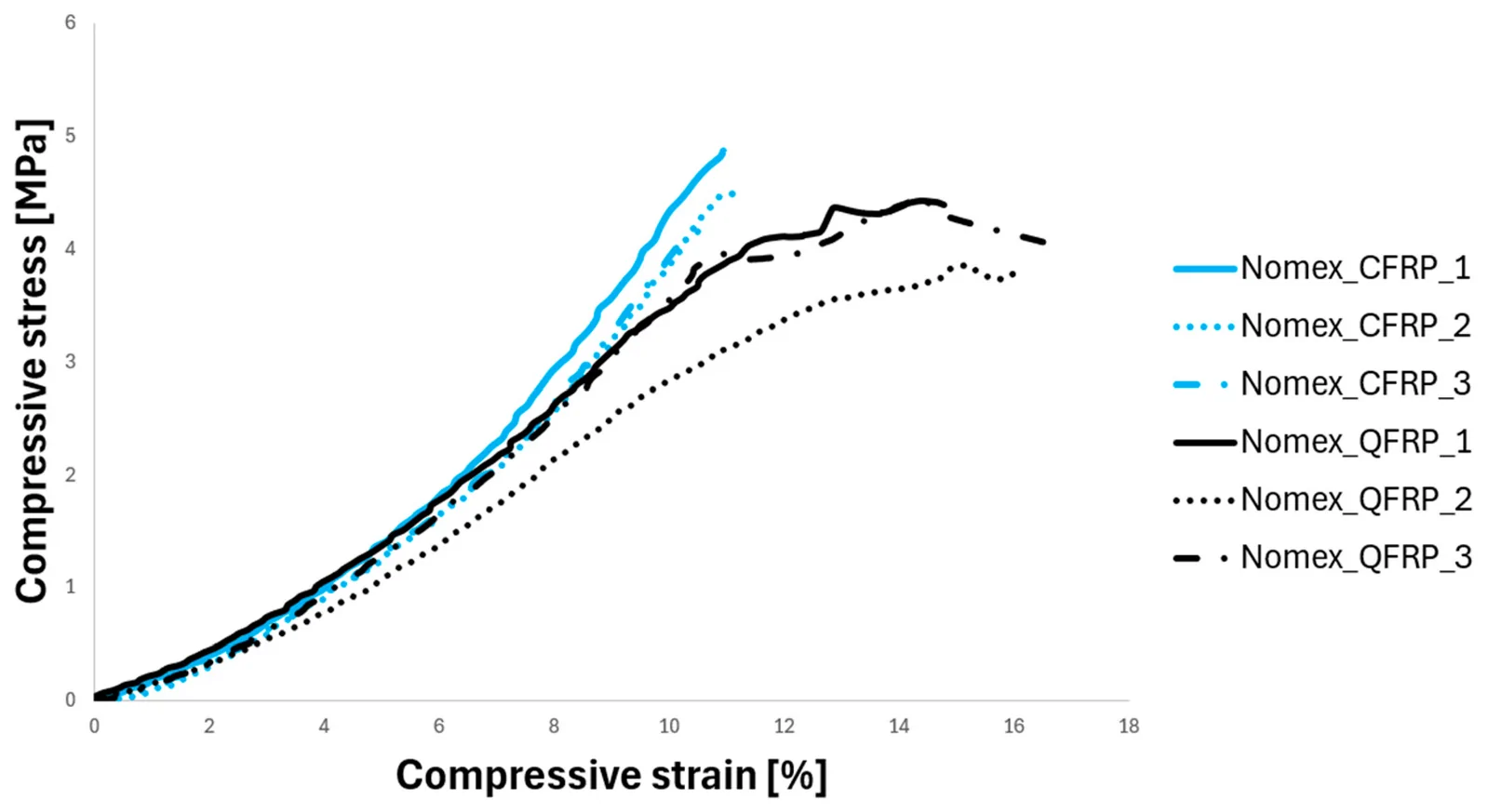
Compression stress–strain curves for Nomex core sandwich configurations, skins: CFRP or QFRP.

Figure 7 highlights the influence of the skin material on the compressive response of the Nomex^®^ sandwich structures. The CFRP-skinned configuration produced higher compressive stresses (4.2–4.9 MPa), but failed at lower strains (≈10%), whereas QFRP skins exhibited slightly lower strengths (3.9–4.5 MPa) together with significantly higher strain-to-failure (14.5–16.5%). The enhanced deformability of the QFRP-skinned panels promoted a more gradual failure process, resulting in smoother post-peak behavior. The higher stiffness of CFRP increased the load-bearing capacity; however, failure occurred abruptly once the compressive strength limit was reached, leading to rapid crushing of the Nomex^®^ core.

**Figure 8:**
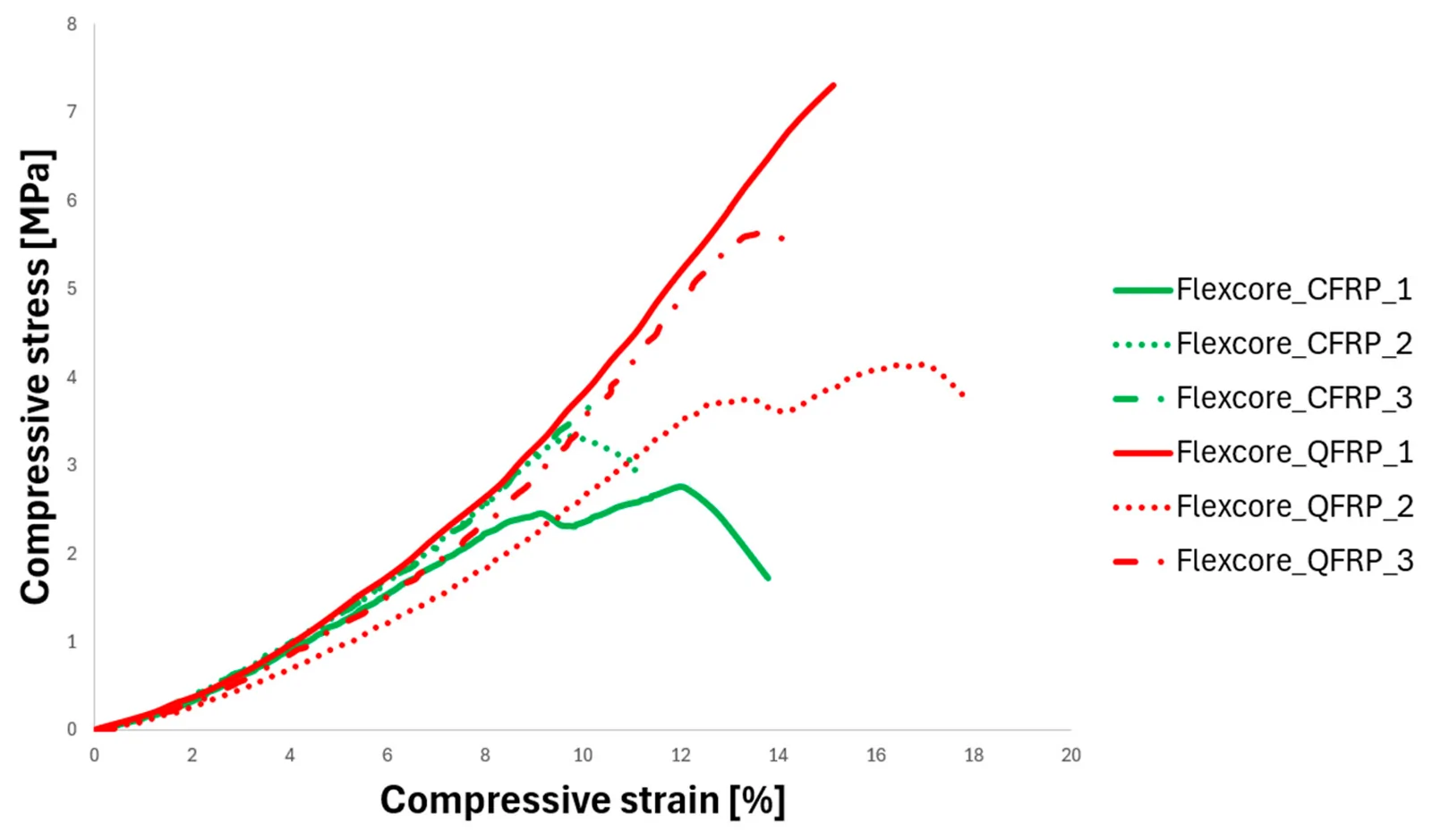
Compression stress–strain curves for Flex-Core^®^ sandwich configurations, skins: CFRP or QFRP.

Figure 8 presents the corresponding compressive response of the Flex-Core^®^ sandwich structures. The QFRP-Flex-Core^®^ configuration exhibited higher maximum compression stress and strain than the CFRP-Flex-Core^®^ configuration. The CFRP-Flex-Core^®^ specimens reached maximum compression stresses of approximately 2.7–3.7 MPa, whereas the QFRP-Flex-Core^®^ specimens reached values between 4.0 and 5.6 MPa. The QFRP-Flex-Core^®^ configuration also sustained a greater compressive deformation before failure. In the Flex-Core^®^ configuration, the compressive curves typically exhibited two stress peaks. QFRP skin sandwich outperforms the CFRP skin sandwich in both maximum stress and strain, highlighting how the core material’s properties dictate which face sheet performs better.

**Figure 9:**
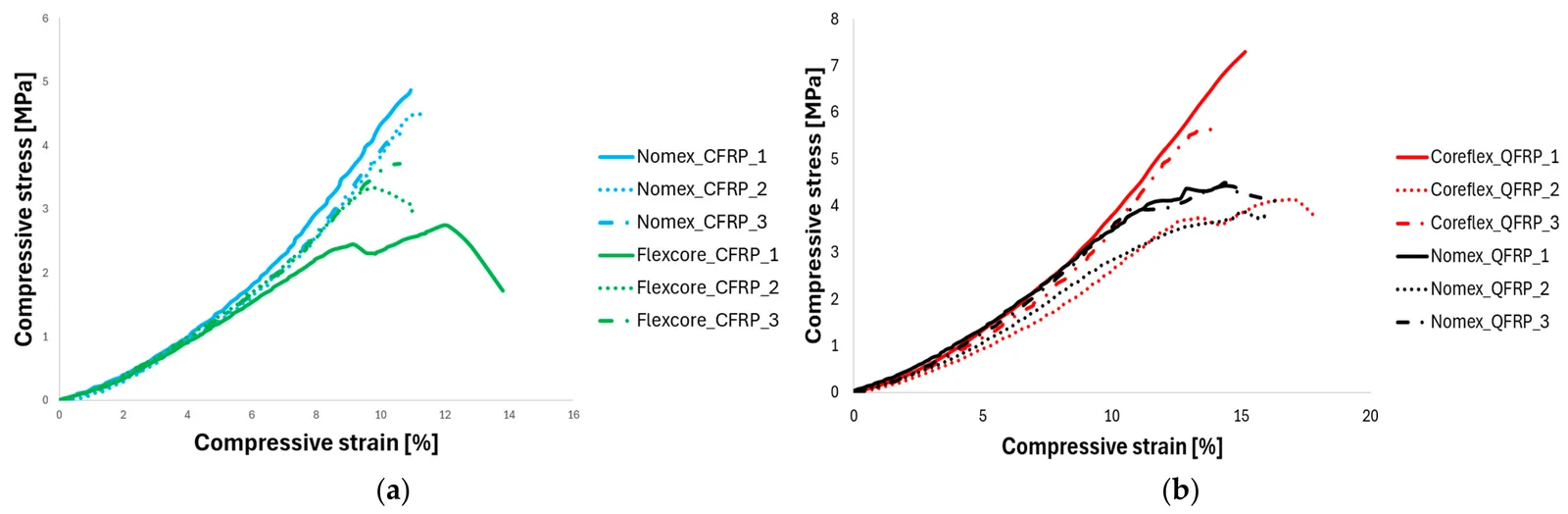
Compression stress–strain curves for (**a**) CFRP skin sandwich configurations (Flex-Core^®^ or Nomex core); (**b**) QFRP skin sandwich configurations (Flex-Core^®^ or Nomex core).

The impact of core features on the compression behavior is outlined in Figure 9. CFRP skin Nomex core sandwich structures exhibit a higher compression stress (mean value of 4.5 MPa) when compared to CFRP skin Flex-Core^®^ sandwich structures (mean value of 3.2 MPa). Conversely, for QFRP-skinned structures, the Flex-Core^®^ configuration exhibited a higher maximum compression stress (up to 5.6 MPa) than the QFRP/Nomex^®^ configuration (up to 4.5 MPa). These results demonstrate that the relative performance of the two core architectures depends on the selected face-sheet material.

Compression strength, modulus, and yield strength from the compression test are displayed in Table 3.

Two configurations stand out following flatwise compressive tests. The higher yield and compression strengths were recorded for the QFRP/Flex-Core^®^ sandwich configuration, followed by the CFRP/Nomex^®^ sandwich configuration with a reduction of 10% in yield strength and 8% in compression strength. Nevertheless, the highest compression modulus was obtained for the CFRP/Nomex^®^ sandwich configuration.

The differences observed between the CFRP- and QFRP-skinned sandwich structures can be attributed primarily to differences in skin stiffness and deformation capacity. For the Nomex^®^ core, the higher stiffness of CFRP increased the load-bearing capacity; however, failure occurred abruptly once the compressive strength limit was reached, leading to rapid crushing of the Nomex^®^ core. This enhanced deformation capacity of the QFRP-skinned panels is attributed to the intrinsic flexibility of the QFRP laminate, which allows greater deformation and elongation prior to ultimate fracture compared to CFRP. The progressive deformation capacity of the QFRP skins allows the sandwich structure to undergo continuous displacement under compressive loading, effectively reducing abrupt brittle fracturing and promoting a sustained energy dissipation mechanism. Specifically, the QFRP skins exhibited a highly progressive failure mode. Due to their compliant nature, the flexible QFRP skins deform synergistically alongside the cell walls of the Nomex^®^ honeycomb core. This coordinated deformation redistributes localized stress concentrations, thereby allowing the core to undergo progressive buckling over an extended displacement period.

For the Flex-Core^®^ configurations, the observed behavior may be attributed to three distinct mechanisms. Firstly, a clear structural synergy between the face sheets and the Flex-Core^®^, whereby the compatibility between their stiffness and deformation characteristics may influence the compressive response. The lower modulus of elasticity of QFRP makes the skin more compliant with the flexible Flex-Core^®^ geometry. Instead of potentially wrinkling prematurely, the QFRP skin can deform more compatibly with the core, promoting a more uniform load distribution and potentially delaying local instability. The elastic matching between the QFRP skin and the pre-curved Flex-Core^®^ cell walls, which collapse through progressive and continuous folding, therefore allows the structure to exploit a greater portion of the core deformation capacity and reach higher ultimate peak stresses.

The second mechanism is related to failure, governed by interfacial shear and core stabilization and not by the tensile/compressive limit of the skin fibers. The QFRP skin deforms progressively under load, which may promote smoother load transfer across the entire core volume, forcing the Flex-Core^®^ to undergo more progressive cell-wall collapse, potentially enabling greater exploitation of the core’s load-bearing capacity. In contrast, stiff CFRP skin begins to compress, forcing localized stress concentrations at the skin-core bond line, causing core shearing. Furthermore, synergistic strain capacity is a third mechanism governing the Flex-Core^®^ sandwich structures. Even though the Flex-Core^®^ wants to compress further, the brittle nature of carbon fiber causes the skins to fracture and delaminate as soon as the local wrinkling waves exceed the ultimate strain capacity of the resin matrix (10 to 14%), while quartz fiber inherently features a high strain-to-failure ratio. Because the QFRP skins are more compliant and exhibit a higher strain-to-failure than the investigated CFRP skins, they may better accommodate deformation of the flexible core before failure, allowing the sandwich panel to undergo the flexible core’s complete deformation cycle and achieve maximum systemic strain.

These observed compression behaviors can be explained by the stiffness mismatch level between the skins and the core, which is a key design parameter for sandwich structures. These observed compression behaviors can therefore be related to the different stiffness and deformation characteristics of the face sheets and core architectures, although the local stress distribution and failure mechanisms at the skin–core interface were not directly characterized in the present study.

To conclude this section, under flatwise compression testing, both sandwich configurations leverage the progressive, continuous folding of the pre-curved Flex-Core^®^ cell walls. However, the skin material heavily influences the initial load distribution and the post-yield behavior. The highly rigid CFRP skins provide a stiffer boundary condition that restricts localized cell wall rotation at the core-skin interface, resulting in a higher initial peak load but sharper stress concentrations. Conversely, the more ductile QFRP skins deform congruently with the compliant core, smoothing out the transition into the crushing plateau and delaying interfacial debonding during extended core collapse.

Nomex core sandwich configurations behave elastically at the beginning of indentation until a critical load is reached, followed by a sharp load drop, specifically for CFRP-Nomex core sandwiches. This behavior may be associated with the abrupt loss of load-bearing capacity accompanying the first local cell-wall fold. Since microscopic examination was not performed, the specific contribution of phenolic resin fracture and local cell-wall buckling cannot be confirmed experimentally. Nomex has a rigid hexagonal cell geometry; thus, under initial indentation, the straight, vertical cell walls compress elastically, building up energy linearly until a critical peak load is reached. Once that critical threshold is passed, the straight walls may undergo sudden local buckling or folding. This sudden collapse of structural stability causes the immediate, sharp drop in the indentation load.

### 3.3. Three-Point Bending Results

In a sandwich structure, the internal core separates the face sheets to increase the cross-sectional moment of inertia, significantly enhancing the sandwich flexural properties. Through the three-point bending test, we further analyzed the failure mode of the sandwich panel and the factors affecting the flexure properties. Under the three-point bending test of a sandwich panel, the top skin experiences compression, the bottom skin experiences tension, while the core handles shear.

**Figure 10:**
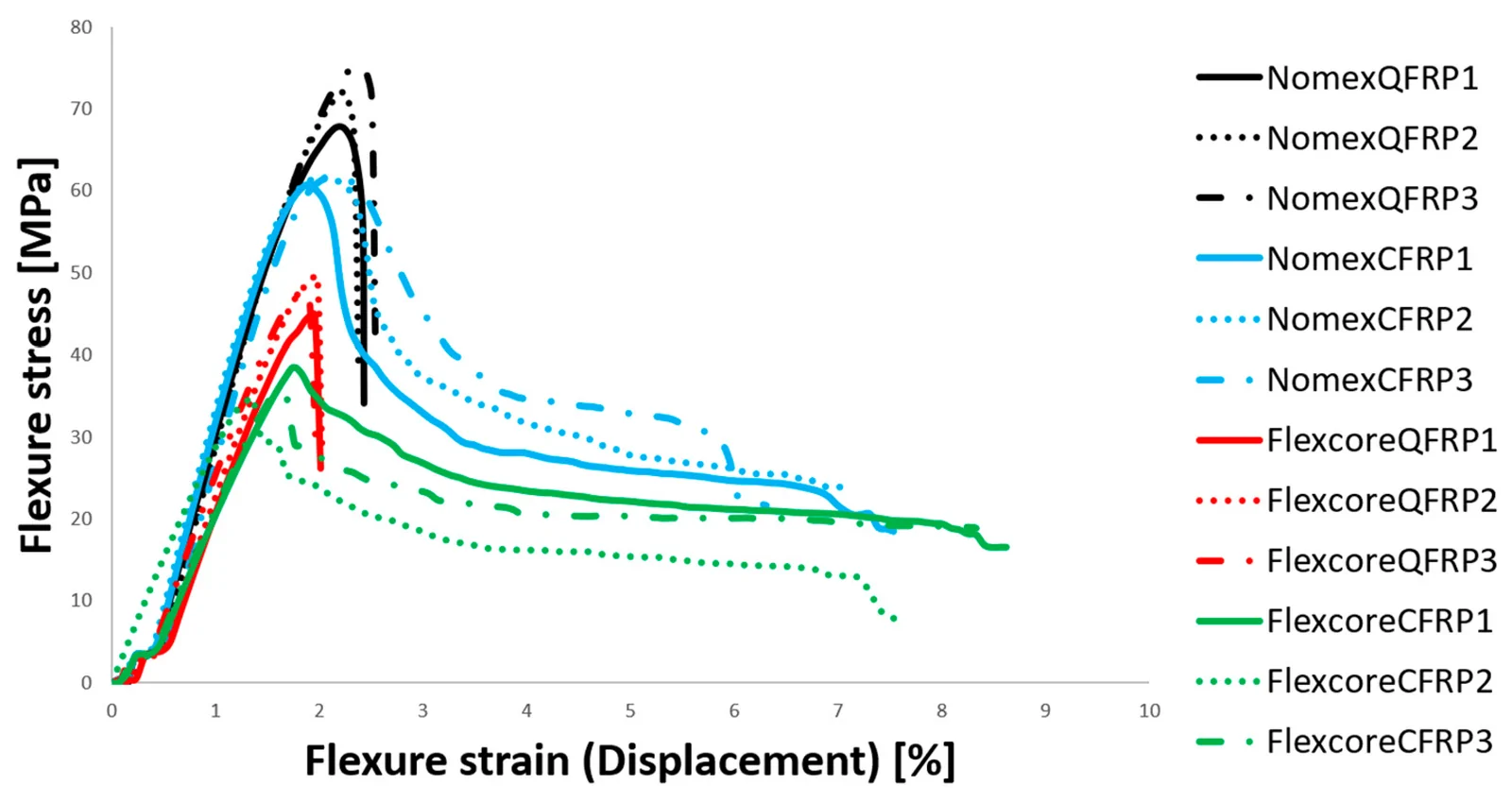
Flexural stress–displacement curves output from 3-point bending sandwich specimen tests.

Figure 10 shows the flexural stress–displacement curves of all 3-point bending tests. The analyzed sandwich structures’ behavior changes radically from compression to flexural tests. The higher flexural stress was obtained for the QFRP/Nomex^®^ sandwich configuration (mean value around 72 MPa), followed closely with only 14% decrease by CFRP/Nomex^®^ sandwich configuration.

The core architecture effect on the flexural behavior of sandwich specimens is important, since passing from Nomex^®^ to Flex-Core^®^, the sandwich specimens’ flexural stress decreases significantly by 34% for QFRP skins and 41% for CFRP skin sandwich specimens, while the flexural strain reduction is negligible for QFRP skins (from 2.4% to 2%) and around 16% for CFRP skin sandwich specimens.

**Figure 11:**
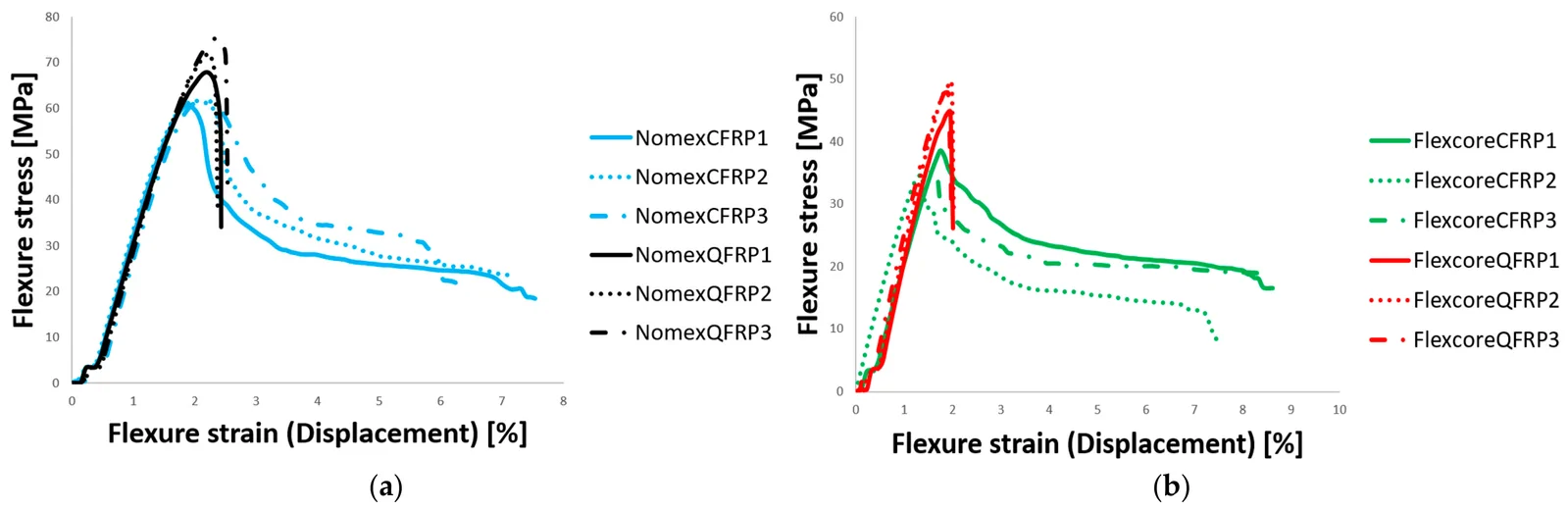
Flexural stress –displacement curves for: (**a**) Nomex^®^ core sandwich and (**b**) Flex-Core^®^ sandwich for both CFRP and QFRP skin configurations.

The skin effect on the flexural behavior of sandwich specimens’ analysis (Figure 11) indicated that for Flex-Core^®^ sandwich configurations, when changing skin from QFRP to CFRP, the flexural stress decreases by 23%. Nevertheless, a significant increase from 2 to 8% in flexure strain is recorded. Referring to Nomex^®^ sandwich configurations, when changing skin from QFRP to CFRP, the flexural stress decreases by 14%, whereas flexure strain increases from 2.4 to 7%.

Although the QFRP/Nomex^®^ sandwich configuration exhibits the higher flexural stress, when looking at the energy at break or the flexural modulus, it stands out that the CFRP/Nomex^®^ configuration sandwich structure absorbs 60% more energy before total structural failure occurs than QFRP/Nomex^®^. Overall, CFRP skin configurations exhibited higher energy at break when compared to QFRP skin configurations (e.g., QFRP/Flex-Core^®^ sandwich configurations reported an 80% decrease). Table 4 clearly underlines that high-stiffness CFRP skin suppresses premature localized failure, driving the integrated Nomex^®^ core into a regime of high-stress progressive cell-wall buckling and shearing over a broader deformation range, maximizing energy dissipation before ultimate collapse, confirmed by the higher strain to break levels.

Cell geometry and stiffness are key factors determining the flexural performance. While regular hexagonal Nomex^®^ cores provide the high vertical and shear stiffness necessary for optimal bending resistance, the over-expanded, curved-wall geometry of Flex-Core^®^ sacrifices flatwise and shear properties. This trade-off occurs because the modified architecture is optimized for formability around complex curvatures without localized cell wrinkling, rather than for structural load-bearing capacity. Additionally, skin compatibility also plays an important role; since CFRP is much stiffer than QFRP, it relies heavily on a highly rigid core to prevent localized failure (like skin wrinkling or core shearing). Thus, when paired with the more compliant Flex-Core^®^, the ultra-stiff carbon skins cannot reach their full structural potential before premature failure occurs.

**Figure 12:**
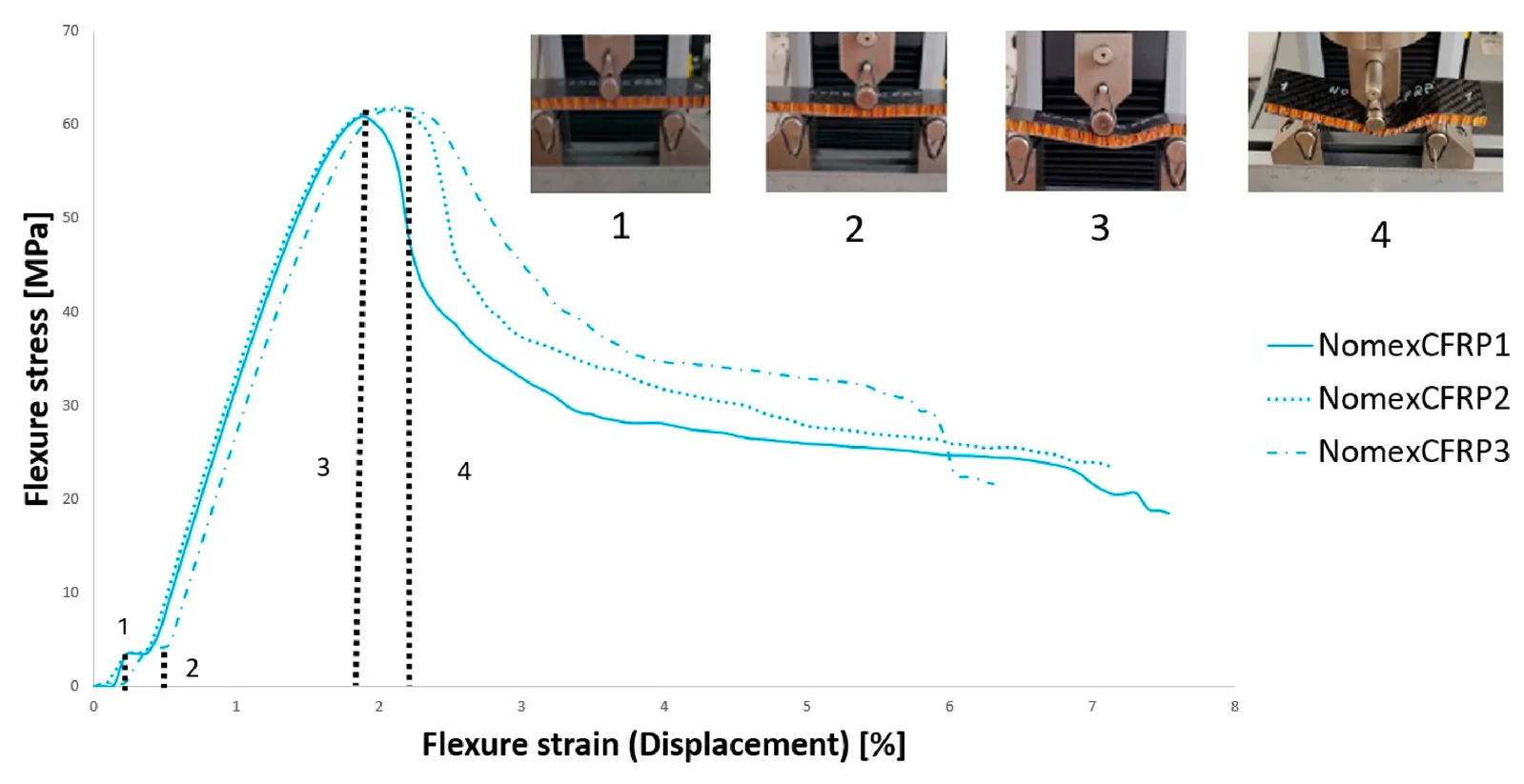
Representative flexural stress–displacement curve and associated failure sequence of the Nomex^®^/CFRP sandwich structure during three-point bending.

Figure 12 displays the failure mode of Nomex/CFRP sandwich bending specimens correlated with the experimental phenomenon.

The bending process of a CFRP Nomex honeycomb sandwich can be described in five stages:

(1) The elastic deformation stage. The sandwich structure exhibits linear elastic behavior, with the load increasing linearly with displacement. The top and bottom CFRP skins absorb normal tensile and compressive stresses, while the Nomex honeycomb core resists the transverse shear forces. No damage occurs during this phase.
(2) The yielding stage. Displacement increases, creating a narrow plateau. Mechanical energy is consumed to buckle or crush that localized layer: local instability of the top CFRP skin may develop under the loading nose.
(3) The stacking stage. Force and displacement increase. The honeycomb walls begin to fold, with the fold lines spreading from bottom to top in the middle of the specimens and spreading from top to bottom at the support point.
(4) Core Yielding and Shear Failure Stage (Peak Load). The sandwich structure reaches its ultimate peak load-bearing capacity. As the bending moment increases, the Nomex honeycomb cells undergo progressive deformation and wall buckling, consistent with core shear deformation/failure (core loses its transverse stiffness, transferring severe stress concentrations to the adhesive bond layer linked to the lower skin).
(5) Debonding and final catastrophic failure stage (post-peak) behavior: A sudden and rapid drop in the load occurs as the structure loses its structural integrity. The accumulation of stress causes rapid interfacial debonding (delamination) between the CFRP face-sheets and the Nomex core. The post-peak response is associated with a loss of structural integrity, potentially involving skin–core interfacial damage, fiber breakage, and matrix cracking.

To conclude this section, Nomex/QFRP achieves the highest maximum flexural stress despite exhibiting a much lower strain, revealing a fundamental shift in the sandwich structure’s failure mechanism from a strain-limited material failure to a stability-limited structural failure. Despite the fact that in classic laminate theory, QFRP inherently possesses a higher material strain-to-failure than CFRP, in a sandwich structure under bending, strain is not just a function of material elongation, but of geometry and structural deflection. While carbon fiber has higher tensile strength, certain high-purity quartz composites exhibit exceptional ultimate compressive strength, delaying top-skin crushing. The lower tensile/compressive modulus of QFRP allows for a more uniform stress distribution across the Nomex cell walls (elastic matching). It may reduce localized stress concentrations associated with premature skin wrinkling in CFRP. By suppressing early localized buckling, the Nomex/QFRP system remains structurally intact up to a much higher global load. When it finally fails, it does not fail through slow, high-deflection bending (which would show high strain). Instead, it reaches a critical global stress limit where the Nomex core undergoes sudden, explosive shear failure or the skin snaps instantaneously. This may limit the development of large structural deflections, resulting in a low recorded strain at the moment of failure. Considering the high stiffness deflection limit, CFRP has a much higher Young’s modulus than QFRP. This high stiffness means the CFRP skin forces the sandwich panel to remain rigid, generating rapid stress buildup at very low deflections. Furthermore, Nomex honeycomb core is cellular; the thin, ultra-stiff CFRP skins experience intra-cell dimpling. Since CFRP is highly rigid, it cannot conform to micro-stresses, reaching its critical wrinkling stress threshold early, causing the skin to buckle or de-bond suddenly at a low structural strain. Flex-Core^®^ sandwich configurations, regardless of the skin type, underperform in maximum flexural stress because they lack the structural geometric boundaries of Nomex. However, when changing the skin from QFRP (recoding higher flexural stress, mean value 47 MPa) to CFRP skin (lower flexural stress, mean value 32 MPa), there is a significant decrease in flexure strain from 8.6% to 2%. A similar trend was observed in Nomex^®^ core sandwich configurations.

### 3.4. DMA Measurements

**Figure 13:**
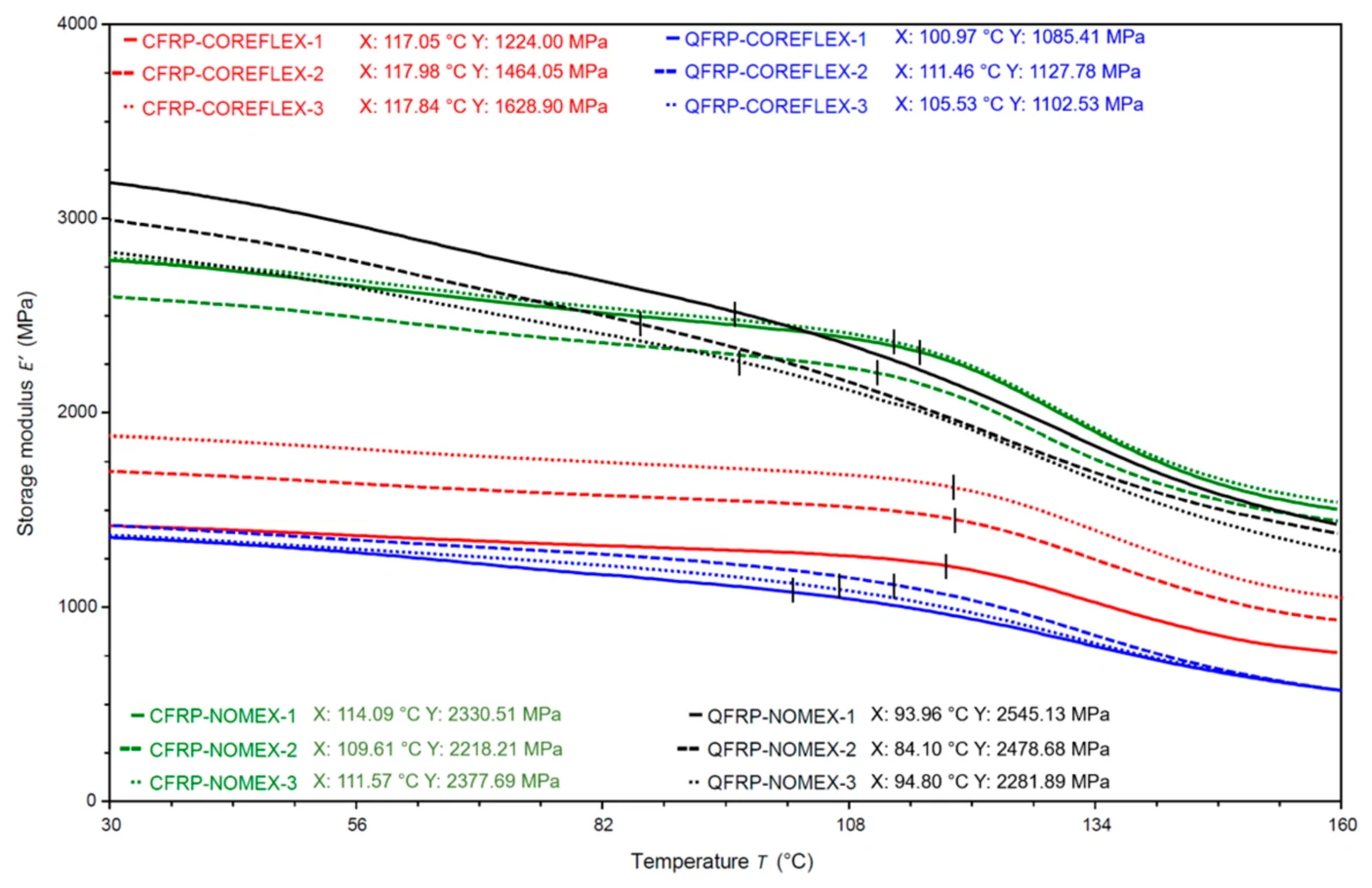
Storage Modulus (E′) curves of analyzed sandwich configurations.

**Figure 14:**
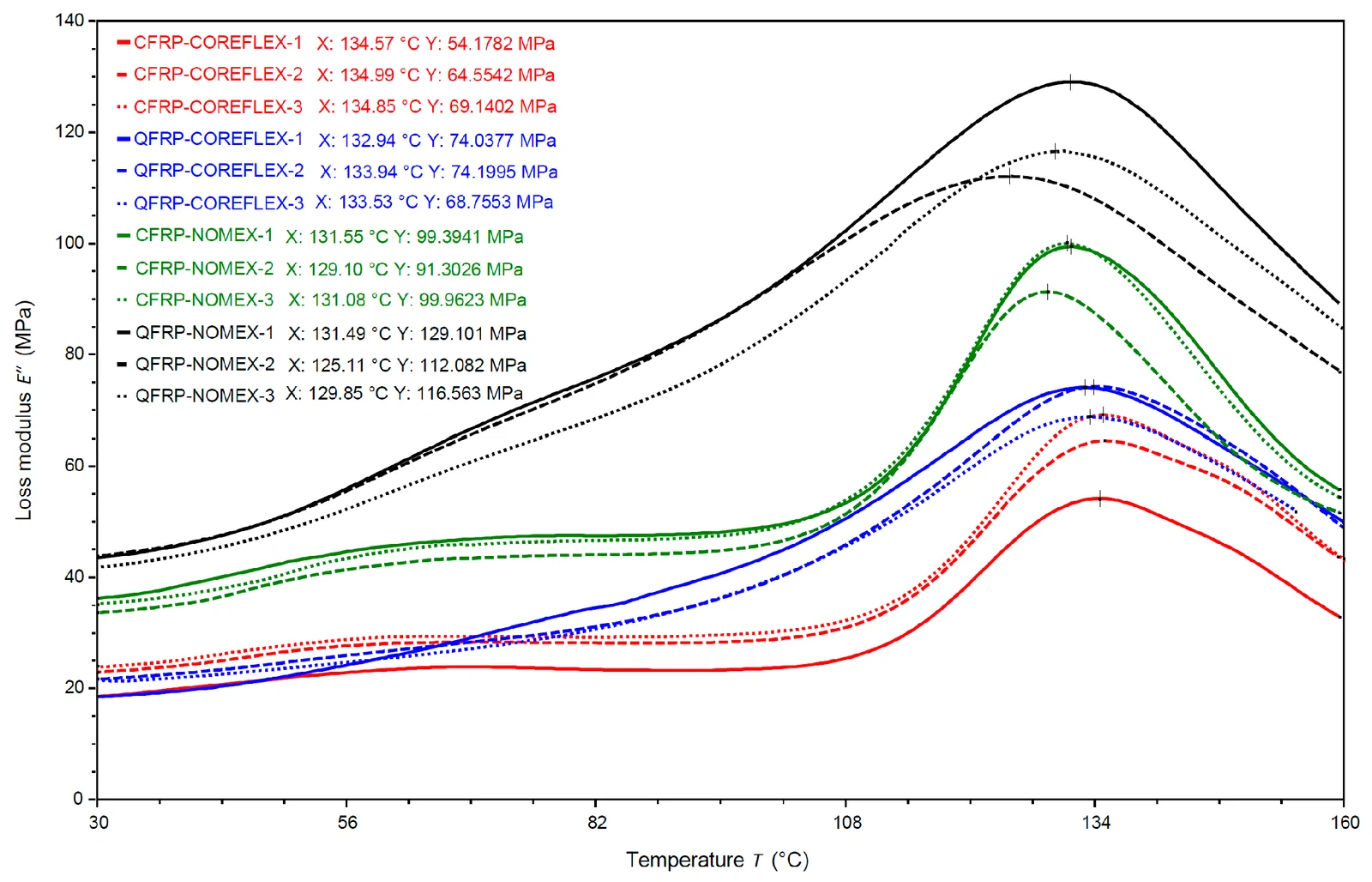
Loss Modulus (E″) curves of analyzed sandwich configurations.

As reported earlier, DMA analysis was performed in a 3-point bending mode at an oscillation frequency of 1 Hz and a maximum force of 18 N, to accommodate the sandwich cross-section’s high moment inertia. Unlike homogeneous materials, sandwich panels behave as structural systems; therefore, the thermo-mechanical response represents the combined contribution of the skins, core, and adhesive interfaces, providing valuable information on stiffness retention, damping behavior, and interfacial integrity. The high-temperature DMA sweeps for the E′ and E″ curves (Figure 13 and Figure 14) do not return to zero, indicating that the material maintains a residual baseline at elevated temperatures. The storage modulus (E′) was primarily governed by the reinforcing fibers within the skins, while the overall response also depended on the core topology (Figure 13).

Nevertheless, regardless of the skin type, all Flex-Core^®^ sandwich configurations exhibited a significant decrease in storage modulus compared with the Nomex-Core^®^ configurations, amounting to 38% for CFRP and 54% for QFRP. Flex-Core has pre-curved, bell-shaped cell walls designed for easy forming; thus, its out-of-plane shear modulus is structurally lower and more compliant than standard hexagonal cells. Since the investigated phenolic honeycomb cores retain structural stability over a broad temperature range, the observed decrease in storage modulus at elevated temperature should not be interpreted directly as a glass transition of the core material. As the temperature rises toward 160 °C, Flex-Core panels show a faster drop in storage modulus compared to a standard rigid Nomex hexagon. For identical Flex-Core^®^ geometries, CFRP skins exhibited approximately 23% higher stiffness than QFRP skins, while in both systems the reinforcing fibers continued to bear the applied load, maintaining the structural stiffness (E′) well above zero.

All sandwich configurations exhibited a pronounced increase in loss modulus (E″) above 130 °C (Figure 14).

CFRP skin sandwich configurations exhibit an identical morphology of the curve, E″, followed by a relaxation around 108 °C and a sharp peak near 135 °C. In contrast, the QFRP configurations exhibited a continuous increase in E″ up to approximately 130 °C. The highest loss modulus values were recorded for the QFRP/Nomex core sandwich configurations (~120 MPa), followed by the CFRP/Nomex configurations (~100 MPa). The QFRP skin/Nomex core configuration exhibits a higher baseline, along with the largest area and highest peak magnitude prior to the main transition, compared to all other sandwich configurations.

**Figure 15:**
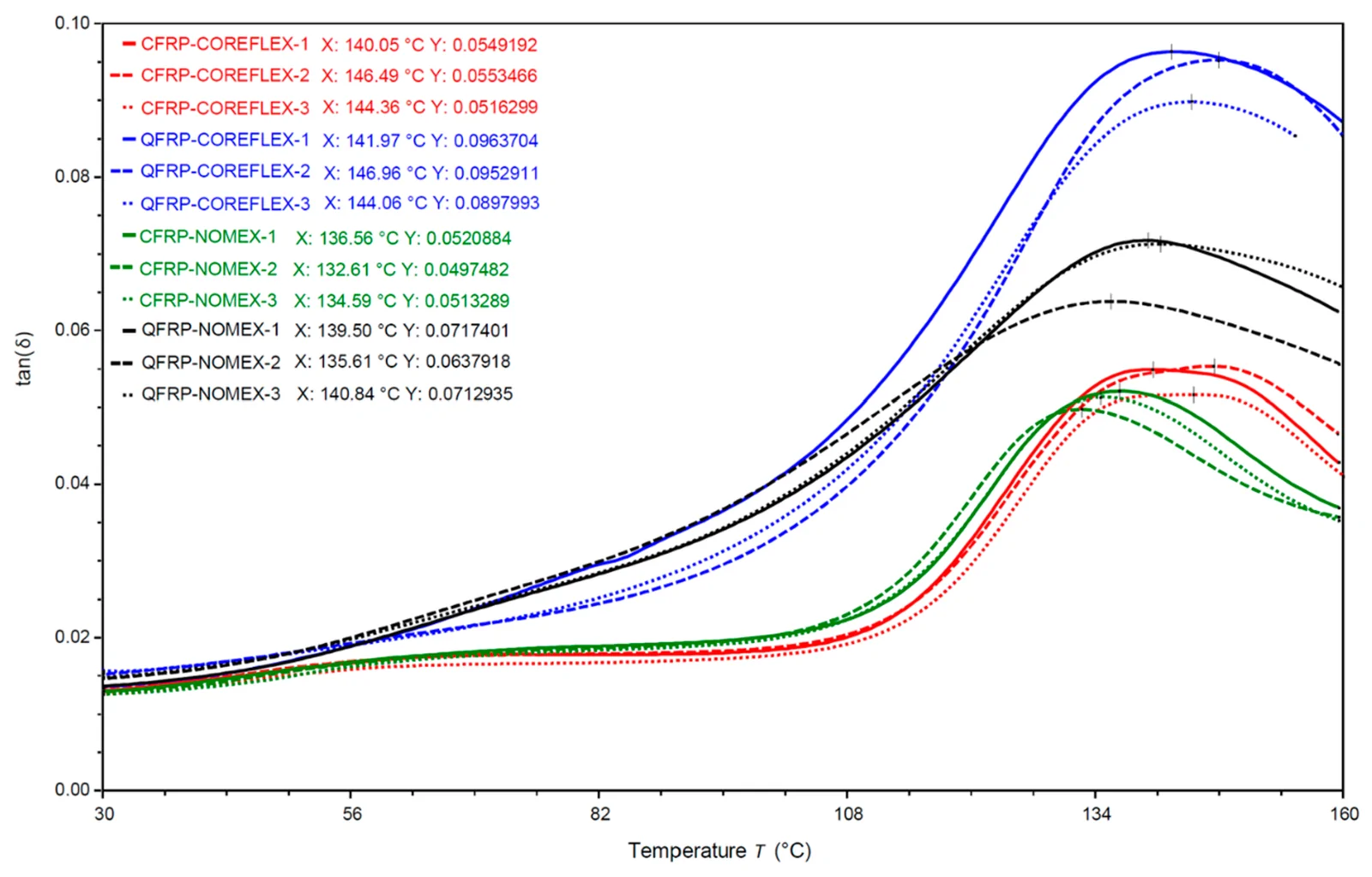
DMA damping profiles (tan δ) of analyzed sandwich configurations.

Dynamic Mechanical Analysis revealed distinct Tan Delta (tan δ) damping profiles during the initial phase of the thermal ramp (as shown in Figure 15).

QFRP/Nomex exhibited a higher damping factor (0.070 at 138 °C) than CFRP/Nomex (0.050 at 134 °C). When analyzing the multi-phase loss modulus and tan δ damping dynamics, we observe that the interaction between mechanical stress fields and thermal transitions becomes apparent when examining the early onboarding stage of the glass transition between 130 °C and 140 °C. As mentioned above, within this narrow thermal window, the damping factor rises for both CFRP and QFRP skins when transitioning from a Nomex^®^ to a Flex-Core^®^ core. The core effect on sandwich structures’ damping behavior was also noticeable. Replacing the Nomex^®^ core with Flex-Core^®^ increased tan δ for both skin systems, from 0.050 to 0.055 for CFRP and from 0.070 to 0.090 for QFRP (28% increase). Since tan δ = E″/E′, the decrease in E′ (38% to 54% drop) directly contributes to the higher damping values. When the denominator (E′) drops, the overall tan δ ratio automatically spikes upward. Nevertheless, the highest damping factors were consistently recorded for the QFRP sandwich configurations with either Nomex^®^ core or Flex-Core^®^.

In thermosetting systems like epoxy/cyanate ester co-networks, this behavior confirms that the material does not undergo complete macromolecular relaxation or transition into a fully liquid state. Instead, it enters a stable rubbery plateau. For the sandwich panel assembly, this residual stiffness prevents catastrophic structural collapse under thermal load, demonstrating excellent high-temperature load-bearing capacity and thermal stability. The polymer matrix (e.g., cyanate ester or epoxy) dictates the baseline thermal limits, while the reinforcing fiber morphology governs the magnitude of the moduli curves. The CFRP/Flex-Core^®^ configurations exhibited the highest initial storage modulus owing to the higher tensile modulus of carbon fibers (~200 GPa), whereas the QFRP/Flex-Core^®^ configurations displayed a lower baseline due to the lower tensile modulus of high-purity silica quartz fibers (~70 GPa). The faster drop that can be seen in Figure 12 for the Flex-Core panels may be related to their greater geometric compliance rather than to constituent-level thermal softening alone.

The increase in storage modulus E″ in Figure 13 indicates enhanced energy dissipation during thermal loading. At lower temperatures, CFRP skin sandwich configurations displayed a low E″ because the glassy epoxy matrix restricted molecular mobility, and near 135 °C they showed a sharp peak, potentially due to the epoxy matrix chains sliding past one another, internal friction spiking dramatically. In contrast, the continuous increase in E″ up to 135 °C for the QFRP configurations can be explained as a consequence of the Thermal mismatch between the skins and cores, which may also contribute to localized interfacial sliding and micro-damage during heating.

This behavior may be associated with differences in the viscoelastic response of the QFRP skin, fiber–matrix system, adhesive and core interfaces. Quartz fibers typically have a different surface chemistry and roughness compared to carbon fibers. The higher loss modulus indicates greater energy dissipation at the structural level, although the individual contribution of the skin–core interface cannot be isolated from the present measurements. Under dynamic loading, the polymer chains at the QFRP interface experience more resistance to movement, leading to greater mechanical damping. Conversely, the higher stiffness of carbon fibers forms a narrower, sharper interphase, restricting matrix deformation and internal friction, resulting in a lower loss modulus.

Furthermore, although a sharp peak was observed for the QFRP skin sandwich configurations around 130 °C, this transition cannot be uniquely assigned to the cyanate ester matrix or the phenolic dipping resin based on the present sandwich-level DMA measurements. The observed response represents the combined behavior of the skins, adhesive, and core.

The skin effect on how effectively the sandwich structure can absorb and dampen vibrations is obvious. Because of the greater viscoelastic energy dissipation of the quartz-fiber skins, the QFRP/Nomex displayed a higher damping factor. When analyzing the multi-phase loss modulus and tan δ damping dynamics, we observe that the interaction between mechanical stress fields and thermal transitions becomes apparent when examining the early onboarding stage of the glass transition between 130 °C and 140 °C. As mentioned above, within this narrow thermal window, the damping factor rises for both CFRP and QFRP skins when transitioning from a Nomex^®^ to a Flex-Core^®^ core. This systematic shift toward higher viscoelastic energy dissipation validates the storage modulus knockdown; the geometric compliance of the curved Flex-Core^®^ cell walls accelerates structural yielding under shear loads, elevating internal friction prior to the chemical softening of the skins.

The increase in tan δ for Flex-Core^®^ compared to Nomex^®^ is attributed to the greater geometric compliance of the curved Flex-Core^®^ cell walls, which promote out-of-plane shear deformation and reduce the global storage modulus. The skins also had an impact on the results, with tan δ recording higher values for the configurations with QFRP skins regardless of the core geometry, indicating that energy dissipation is primarily governed by the viscoelastic response of the skin material.

Notably, post-test visual inspection revealed no macroscale damage, delamination, or structural failure in either the QFRP or CFRP skins sandwich configurations. This confirms that the oscillatory load of 18 N at 1 Hz remained safely within the linear viscoelastic region of the constituent materials, ensuring non-destructive evaluation across the investigated temperature spectrum.

Tg is critical for structural design as it marks the onset of stiffness loss. The tan δ peaks occurred at 135–138 °C for Nomex configurations and ~143 °C for Flex-Core^®^ configurations, showing no correlation with the skin polymer Tg. Although low due to the lack of post-curing, these values reflect system-level properties where E′ drops artificially due to core softening or adhesive degradation. Consequently, the observed tan δ peaks should be considered system-level thermo-viscoelastic transitions rather than direct measurements of the Tg of a specific constituent. Independent characterization of the individual skin, adhesive, and core materials would be required for definitive assignment of these transitions.

## 4. Conclusions

Static mechanical tests and DMA analysis performed on the aramid core developed sandwich structures revealed the following conclusions:

1. QFRP/Nomex sandwich configurations exhibit the highest flexural stress despite displaying a much lower strain, while CFRP/Nomex sandwich configurations recorded a slightly lower flexural stress (14%), whereas flexure strain increased from 2.4 to 7%. DMA measurements showed a similar trend, as it can be observed that the highest E′ storage modulus for QFRP/Nomex (mean value 2435 MPa) was followed by CFRP/Nomex with a 100 MPa decrease (mean value 2308 MPa). The loss modulus E″ curves depict a similar trend, with QFRP/Nomex (mean value 120 MPa) followed by CFRP/Nomex with a 20% reduction (mean value 97 MPa).
2. QFRP/Flex-Core^®^ sandwich configurations excel in compression, presenting the highest yield and compression strengths, followed by the CFRP/Nomex^®^ sandwich configuration with a reduction of 10% in yield strength and 8% in compression strength, although recording the highest compression modulus. DMA analysis damping profiles (tan δ) support these findings, since QFRP/Nomex exhibited a higher damping factor (0.070 at 138 °C) than CFRP/Nomex (0.050 at 134 °C), indicating greater viscoelastic energy dissipation at the structural level. The damping factor rises for both CFRP and QFRP skins when transitioning from a Nomex^®^ to a Flex-Core^®^ core, underlining the core architecture effect. The steady shift toward greater viscoelastic energy dissipation confirms the reduction in the storage modulus. The geometric compliance of the curved Flex-Core^®^ cell walls accelerates structural yielding under shear loads, which may increase structural energy dissipation before pronounced thermal softening of the constituent materials.
3. The core architecture effect on sandwich performance was observed in both static mechanical tests. 3-point bending performance is governed by a combination of core shear strength, core modulus, compression/tension strengths of skins (upper/bottom), and the overall interaction of the structural components; thus, QFRP/Nomex^®^ sandwich exhibited the highest measured flexural performance. Flatwise compression tests rely more on the core (density and cell configuration), since the stiffness of CFRP or QFRP skins simply distributes the load; therefore, the QFRP/Flex-Core^®^ sandwich outperformed the other configurations, which may be associated with the different stiffness and deformation characteristics of the skin-core combinations: elastic matching QFRP/Flex-Core^®^ and stiffness matching CFRP/Nomex.
4. Stiffness mismatch level between the skins and the core, which proves to be a key design parameter of sandwich structures development for specific applications. The results indicate that the relative stiffness and deformation characteristics of the skin and core are important design parameters for sandwich structures intended for specific loading conditions.
5. While carbon fiber reinforced polymer (CFRP) skins bonded to Nomex^®^ honeycomb cores represent the industry standard for lightweight sandwich structures, emerging aerospace applications require multi-functional materials such as radar-transparent quartz fiber reinforced polymer (QFRP) skins and flexible FlexCore^®^ matrix systems. However, existing literature predominantly evaluates these advanced material systems in isolation or focuses strictly on the formability of FlexCore^®^ in curved geometries. Consequently, there remains a distinct literature gap regarding a direct, systematic comparison of the flat-plane mechanical response, stiffness efficiency, and failure progression when cross-combining these specific skin (CFRP vs. QFRP) and core (Nomex^®^ vs. FlexCore^®^) architectures. This study fills this gap by conducting a comprehensive comparative analysis of flat sandwich panels manufactured with four distinct configurations: CFRP-Nomex^®^, CFRP-FlexCore^®^, QFRP-Nomex^®^, and QFRP-FlexCore^®^. By subjecting these panels to 3-point bending, flatwise compression, and DMA testing under identical environmental and processing conditions, the effects of skin system and cell geometry were comparatively evaluated.

Ultimately, this work establishes a critical mechanical baseline and selection framework for engineers balancing structural weight, core geometry, and electromagnetic requirements in aerospace design.

## Figures and Tables

**Table 1:** Specifications of the cores used to develop the present study sandwich structures.

Feature	Core Type	
	Nomex HRH-10-3.2-64	HexWeb^®^ Nonmetallic Flex-Core^®^ HRH10-F50
Material	Nomex aramid paper dipped in phenolic resin aramid HRH10 aero grade	
Cell geometry	Hexagonal	Unique curved, corrugated cell-bell shape
Density	64 kg/m^3^	56.1 kg/m^3^
Thickness	6 ± 0.1 mm	6 ± 0.1 mm
Cell distortion	Wall buckling	None (bell-shaped cell walls open and close fluidly to match contours-ensuring uniform sandwich mechanical properties)
Structural Integrity	Affected (pre-stressed and damaged cells- thermoforming)	Preserved (cells are not pre-stressed or structurally compromised during lay-up; the final component maintains its full impact resistance and skin-to-core shear strength across the entire curved surface)
Flexibility	Highly rigid, prone to tearing on tight radii	Drapes well over compound curves
Core Spring-Back effect	Lift out of tight corners, creating unbonded voids between the skin and the core	Stays flat against the mold, ensuring a high-quality bond line
Manufacturing (complex shapes)	Need CNC machining or thermoforming (high costs of Labor and Tooling)	Hand-layer directly into the complex mold
Applications	High anti-crushing strength and excellent shear properties along the ribbon direction (L), ideal for flat panels, straight aerodynamic fairings	Bends freely in any direction over extreme compound radii without causing cell wall collapse. Ideal for highly contoured surfaces (aerospace and defense, e.g., radomes, nose cones, wing fairings)

**Table 2:** Mechanical tests sandwich specimens’ dimensions.

Sandwich Configuration	Skin Material/ IF Adhesive	Core Material	Sample No.	Sample Dimension Mean Value ± 0.1 mm 3 Samples Tested/Configuration				
				3-Point Bending			Compression	
				Thickness [mm]	Width [mm]	Length/ Span Length [mm]	Thickness [mm]	Length × Width [mm]
CFRP/ Nomex	HexPly^®^ M49/42%/245 g/T2 × 2/ CHS-3k/Toray MicroPly™ TC310	Nomex^®^ HRH-10-3.2-64	1	6.477	30	120/70	6.547	60 × 60
			2	6.463	30	120/70	6.57	60 × 60
			3	6.387	30	120/70	6.537	60 × 60
CFRP/ FlexCore	HexPly^®^ M49/42%/245 g/T2 × 2/ CHS-3k/Toray MicroPly™ TC310	Flex-Core^®^ HRH10-F50	1	6.523	30	120/70	6.640	60 × 60
			2	6.563	30	120/70	6.657	60 × 60
			3	6.587	30	120/70	6.657	60 × 60
QFRP/ Nomex	Toray EX-1515 8HS 4581 AQ III, 330g/m^2^/ MicroPly™ EX-1516	Nomex^®^ HRH-10-3.2-64	1	6.577	30	120/70	6.683	60 × 60
			2	6.663	30	120/70	6.700	60 × 60
			3	6.647	30	120/70	6.710	60 × 60
QFRP/ Flex-Core^®^	Toray EX-1515 8HS 4581 AQ III, 330g/m^2^/ MicroPly™ EX-1516	Flex-Core^®^ HRH10-F50	1	6.720	30	120/70	6.810	60 × 60
			2	6.793	30	120/70	6.790	60 × 60
			3	6.797	30	120/70	6.820	60 × 60

Mean thickness value was reported based on 3-point measurement (middle and 15 mm distance left/right from center) on each test sample, tolerances in thickness max. 2% according to ASTM D7028-7:2015 [22].

**Table 3:** Summary of flatwise compression test results of sandwich structures.

Sandwich Configuration	Sample No.	Compression Modulus (MPa)		Yield Strength (0.2%) [MPa]		Compression Strength [MPa]	
CFRP/Flex-Core^®^	1	33.25	44.23 *	2.41	3.14 *	2.76	3.27 *
	2	46.385		3.33		3.34	
	3	53.05		3.69		3.71	
QFRP/Flex-Core^®^	1	70.92	55.27 **	-	4.64 *	7.3	4.89 **
	2	42.25		3.7		4.14	
	3	68.29		5.58		5.64	
CFRP/Nomex	1	67.85	64.77 *	-	4.18 *	4.87	4.52 *
	2	62.23		-		4.5	
	3	64.23		4.18		4.19	
QFRP/Nomex	1	46.20	45.27 *	3.75	3.54 *	4.42	4.26 *
	2	37.54		2.91		3.88	
	3	52.06		3.95		4.49	

* mean values. ** QFRP/Flex-Core^®^-1 was not considered.

**Table 4:** Summary of 3-point bending results of sandwich structures.

Sandwich Configuration/ Sample No.		Max. Flexural Stress [MPa]	Flexural Stress at Break [MPa]	Flex Strain at Break [%]	Energy at Break [J]	Modulus [MPa]
QFRP/Nomex	1	67.9	56.9	2.41	1.38	4662.4
	2	71.9	38.2	2.38	1.34	4869.8
	3	75.3	41.1	2.54	1.56	4774.9
CFRP/Nomex	1	60.8	18.5	7.53	3.45	4896.2
	2	61.6	23.6	7.11	3.59	4933.9
	3	61.8	21.6	6.31	3.33	4836.8
CFRP/Flexcore	1	38.6	16.6	8.62	2.96	3006.6
	2	34.7	7.5	7.56	2.1	3115.9
	3	35.7	18.9	8.33	2.71	3040.4
QFRP/Flexcore	1	44.9	26.2	2.01	0.66	3617.9
	2	49.6	29.2	2.02	0.75	3667.3
	3	47.8	27.9	1.97	0.76	3619.3

## Data Availability

The original contributions presented in this study are included in the article. Further inquiries can be directed to the corresponding author.
